# Testicular Rupture Following Motorcycle Accident

**DOI:** 10.7759/cureus.41609

**Published:** 2023-07-09

**Authors:** Luke W Eldore, Trevor Borries, Hamza Malick, Kiera Mason, Gregory DePrisco

**Affiliations:** 1 Medical School, Texas A&M College of Medicine, Dallas, USA; 2 Diagnostic Radiology, Baylor University Medical Center, Dallas, USA

**Keywords:** testicular hematoma, ultrasound of testicles, ultrasound imaging, ultrasound anatomy, motorcycle accident, doppler ultrasound, testicle, traumatic injury, testicular rupture

## Abstract

Testicular rupture is a surgical emergency necessitating prompt diagnosis and intervention to prevent irreversible damage. Blunt trauma, including motorcycle collisions, is a common cause of testicular rupture. In the case of multi-trauma, the diagnosis of testicular rupture may be missed in the rush to surgical intervention of more grossly obvious injuries. We present a case of a 24-year-old male who suffered a motorcycle accident and subsequently presented with diffuse abdominal and hip pain. Physical exam and imaging at the emergency department showed multiple pelvic bone fractures, along with a small scrotal injury which was triaged below his pelvic injuries. His pelvic fractures were immediately operated on. Nearly 18 hours after his initial presentation, the patient received a scrotal ultrasound which demonstrated a rupture of the right testicle. Due to this long delay in diagnosis, his urological team opted for non-surgical management and instead employed a more conservative treatment regimen involving pain control, scrotal support, rest, and serial scrotal ultrasounds. This case highlights the importance of timely ultrasound examination for testicular pathology in the setting of multi-trauma and known scrotal injury. Another highlight of this case is the showcase of an uncommon treatment regimen utilizing conservative tactics as opposed to opting for surgical intervention.

## Introduction

While an uncommon sequela of trauma, testicular rupture is a surgical emergency that requires prompt diagnosis and management to prevent irreversible damage. Most causes of testicular rupture are associated with blunt trauma such as athletics and motor vehicle accidents [1]. Motorcycle collisions specifically are associated with an increased risk of testicular trauma and rupture [1]. Testicular rupture is characterized as disruption of the surrounding tunica albuginea [2-4]. However, the presentation of symptoms is often non-specific and generalized to the pelvic region, especially in the context of other pelvic injuries, which can result in delayed presentation and mislead diagnostic prioritization [3,5]. Nonetheless, ultrasonography has been found to maintain the highest degree of diagnostic sensitivity and accuracy when evaluating testicular pathologies, including testicular rupture [2,4].

## Case presentation

**Figure 1:**
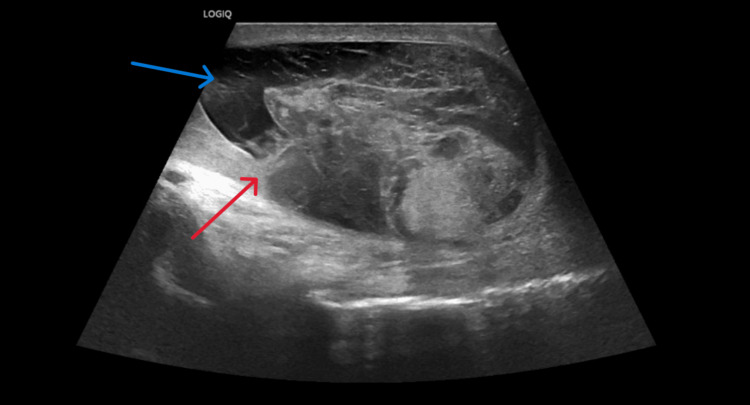
Sagittal grayscale ultrasound of the right testicle approximately 18 hours after injury. Exam shows contour irregularity and tunica albuginea disruption (red arrow), hematoma collection (blue arrow), and heterogenous echotexture. These findings are consistent with a radiological diagnosis of right testicular rupture.

**Figure 2:**
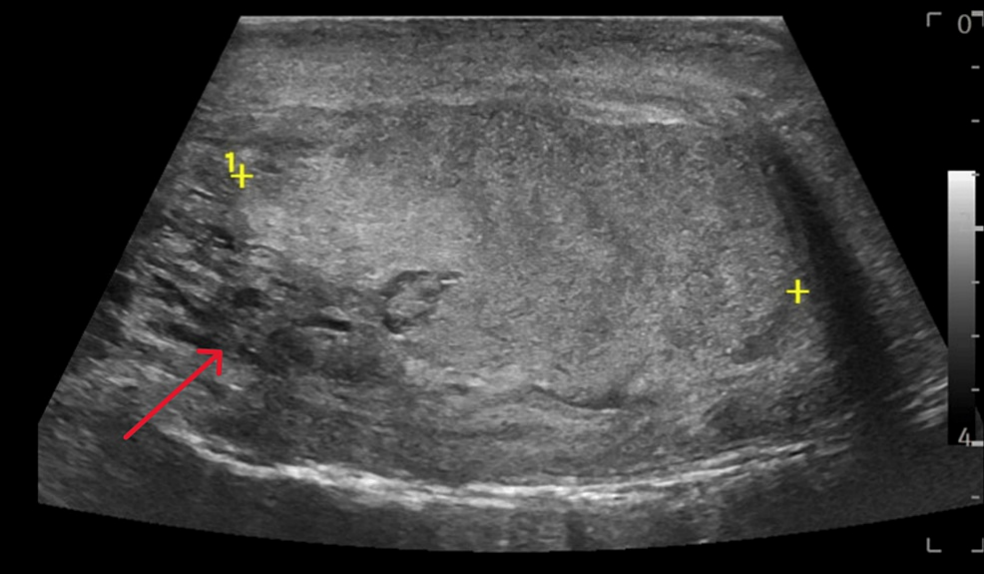
Sagittal grayscale ultrasound of the left testicle approximately 18 hours after injury. Examination shows heterogenous echotexture (red arrow) indicating possible rupture, though continuity of the tunica albuginea appears intact on this series. A diagnosis of testicular rupture was not able to be made based on the findings from this exam.

**Figure 3:**
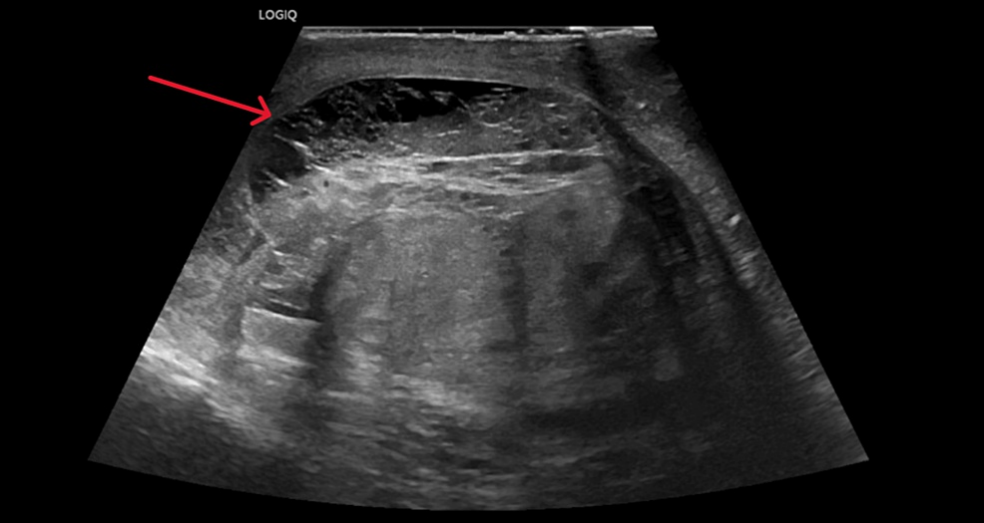
Follow-up sagittal grayscale ultrasound of the right testicle three days after injury. Hematoma is stable from prior examination (red arrow) and heterogenous echotexture are still present on this examination.

**Figure 4:**
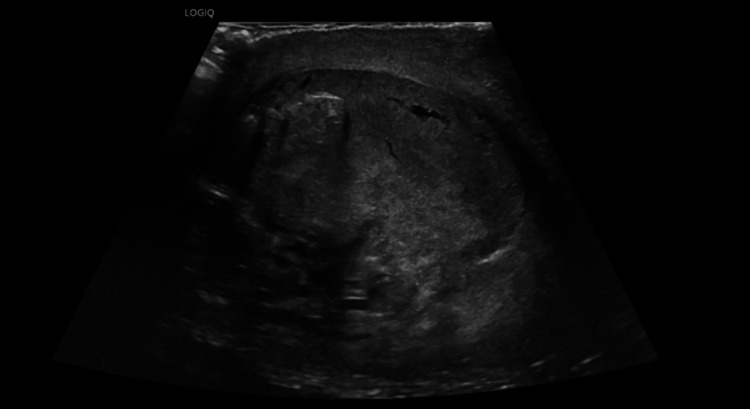
Follow-up sagittal grayscale ultrasound of the left testicle three days after injury. Demonstration of nonspecific heterogenous echotexture that is largely unchanged from the prior study. Testicular contour is normal with a continuous tunica albuginea. This examination was considered stable from the prior ultrasound examination.

A 24-year-old male presented to the emergency department with diffuse abdominal and right hip pain after a motorcycle accident. Physical examination showed an alert male with stable vitals. His hips were unstable to palpation, so a pelvic binder was placed. A small, 1-inch wide laceration was also noted on the right hemiscrotum. Computer tomography (CT) and x-ray evaluation demonstrated multiple fractures including acute, comminuted pelvic fractures which were treated with immediate surgery. After undergoing surgical management of these multiple fractures, attention was turned to the scrotal injury, and a scrotal ultrasound examination was performed. This showed discontinuity of the right tunica albuginea with eccentric echogenicity of the right testicular body and a heterogenous, avascular collection, compatible with right testicular rupture and an associated hematoma (Figure 1, 2). The left testicle was noted to have heterogeneous echotexture without evidence of rupture (Figure 3). The urology team opted for non-operative management of the scrotal trauma due to a nearly 18-hour delay in diagnosis of the testicular injury. Conservative management was employed for this patient, which involved pain control, scrotal support via the use of a towel placed under the scrotum, rest, and serial ultrasounds to monitor for expansion of the hematoma. A follow-up scrotal ultrasound on the following day, however, showed no significant changes (Figure 4). The long-term outcome of this patient is unknown due to the inability to access the patient's records once outside the hospital, though the expectation held by his urological team was that the patient would completely lose function of his right testicle.

## Discussion

This case highlights the importance of prompt ultrasound evaluation of testicular pathology in the setting of multi-trauma and scrotal injury as well as ultrasound monitoring after a treatment plan is made and implemented. Testicular infarction occurs approximately six hours after the onset of ischemia, thus the single most important prognostic factor for testicular salvage is time to surgical intervention [6,7]. Ultrasound findings include testicular contour irregularities, discontinuity in the tunica albuginea, and heterogeneous echotexture of the testis with possible regions of avascularity are characteristic of testicular rupture [2-4,8]. Disruption of the tunica albuginea by itself has a sensitivity and specificity of approximately 50% and 75%, respectively [2].

Testicular rupture is primarily managed surgically with debridement and repair of the tunica albuginea [6,7,9-11]. As mentioned prior, speed of diagnosis is of utmost importance, though with this patient, it was deemed necessary by the trauma team to address his pelvic fractures first before intervening in the patient's testicular injury. This leads to another highlight of this case, which is the showcase of a conservative approach to a known testicular rupture. Conservative management of testicular rupture includes pain control, scrotal support, rest, and serial ultrasounds to evaluate for complications, such as expanding hematoma or expanding testicular infarct. This treatment regimen has been demonstrated to be safe in both pediatric and adult populations, and in this patient, conservative management was also demonstrated to be safe without the development of complications [9-11].

This treatment regimen has been shown in one case series to lead to a better outcome profile as compared to surgical intervention. In that case series, the testicular salvage rate was 100% as there were no cases where patients needed to have an orchidectomy or a surgical exploration [10]. A 2010 paper showed similar findings, where a cohort of seven boys between the ages of 11-18 were managed with a conservative regimen as detailed prior. This study group only had one participant who needed a surgical intervention at a remote later date from the date of his injury, due to the development of a symptomatic hematoma [11]. Interestingly, both of these studies remarked that other capsulated organs, such as the kidneys, are primarily managed non-surgically in the setting of blunt trauma, and argued the testicles should be treated the same. These papers also showed conservation in testicular size with this conservative regimen, though it should also be noted these studies were limited as neither assessed for potential loss of function of the injured testicle [10,11]. Further studies are needed before a conservative treatment regimen for testicular rupture can be widely adopted as the new standard of care.

## Conclusions

Ultrasound evaluation is a valuable tool for the diagnosis of testicular injuries, such as rupture of the testis. Timely diagnosis is needed for surgical intervention, which is still considered to be the standard of care. However, a conservative approach to care may be employed safely in select cases, which is a key highlight of this report.
